# Effect of engineered TiO_2_ and ZnO nanoparticles on erythrocytes, platelet-rich plasma and giant unilamelar phospholipid vesicles

**DOI:** 10.1186/1746-6148-9-7

**Published:** 2013-01-11

**Authors:** Metka Šimundić, Barbara Drašler, Vid Šuštar, Jernej Zupanc, Roman Štukelj, Darko Makovec, Deniz Erdogmus, Henry Hägerstrand, Damjana Drobne, Veronika Kralj-Iglič

**Affiliations:** 1 Biomedical Research Group, Faculty of Health Sciences, University of Ljubljana and Prva-K Klinika za male živali d.o.o. (Prva-K Clinic for Small Animals), Ljubljana, Slovenia; 2 Department of Biology, Biotechnical Faculty, University of Ljubljana, Ljubljana, Slovenia; 3 Laboratory of Clinical Biophysics, Chair of Orthopaedics, Faculty of Medicine, University of Ljubljana, Ljubljana, Slovenia; 4 Biomedical Research Group, Faculty of Health Sciences, University of Ljubljana, Ljubljana, Slovenia; 5 J Stefan Institute, Ljubljana, Slovenia; 6 Department of Electrical and Computer Engineering, Northeastern University, Boston, MA, USA; 7 Department of Biology, Abo Akademi University, Abo/Turku, Finland

**Keywords:** Engineered nanoparticles, Erythrocyte shape, Platelet activation, Thrombosis, Cancer, Dog, Phospholipid vesicles, Biological membrane, Titanium, Zinc oxide

## Abstract

**Background:**

Massive industrial production of engineered nanoparticles poses questions about health risks to living beings. In order to understand the underlying mechanisms, we studied the effects of TiO_2_ and ZnO agglomerated engineered nanoparticles (EPs) on erythrocytes, platelet-rich plasma and on suspensions of giant unilamelar phospholipid vesicles.

**Results:**

Washed erythrocytes, platelet-rich plasma and suspensions of giant unilamelar phospholipid vesicles were incubated with samples of EPs. These samples were observed by different microscopic techniques. We found that TiO_2_ and ZnO EPs adhered to the membrane of washed human and canine erythrocytes. TiO_2_ and ZnO EPs induced coalescence of human erythrocytes. Addition of TiO_2_ and ZnO EPs to platelet-rich plasma caused activation of human platelets after 24 hours and 3 hours, respectively, while in canine erythrocytes, activation of platelets due to ZnO EPs occurred already after 1 hour. To assess the effect of EPs on a representative sample of giant unilamelar phospholipid vesicles, analysis of the recorded populations was improved by applying the principles of statistical physics. TiO_2_ EPs did not induce any notable effect on giant unilamelar phospholipid vesicles within 50 minutes of incubation, while ZnO EPs induced a decrease in the number of giant unilamelar phospholipid vesicles that was statistically significant (p < 0,001) already after 20 minutes of incubation.

**Conclusions:**

These results indicate that TiO_2_ and ZnO EPs cause erythrocyte aggregation and could be potentially prothrombogenic, while ZnO could also cause membrane rupture.

## Background

With increasing industrial production of engineered nano and microparticles, questions have been raised on their effects on humans and animals [1-4]. Engineered particles enter the body by inhalation [5], ingestion [6] and by corrosion of implants [7,8], while possibilities of injection of engineered nanoparticles for imaging and therapeutic purposes are being explored [9,10]. In order to protect ourselves from their (potentially) harmful effects and use them beneficially, their interactions with living beings should be well studied and the underlying biochemical and biophysical mechanisms which take place on the mesoscopic and nanoscopic level [11-17] should be better understood.

It was observed that engineered nanoparticles can cross the plasma membrane to be internalized in cells where they may cause apoptosis [18] and interact with proteins [19]. It is therefore indicated that studying the interaction of engineered nanoparticles with membranes [20-22] is important for the benefit of human and animals. These studies do not require experiments on laboratory animals or materials derived from them. Engineered nanoparticle-membrane interactions can be studied by focusing on nonspecific biophysical mechanisms that are common in different types of biological membranes, i.e. on uni- and multilamelar lipid vesicles or selected cell models [20,23,24]. Giant unilamelar phospholipid vesicles larger than 1 μm (GUVs) [25-28] are a convenient model system as they can be observed in motion under a light microscope and enable studies of biological membrane structure [22], phase behaviour [29,30], permeability [31,32] and elasticity [33,34], as well as of their interaction with macromolecules [35]. Another suitable model is represented by mammalian erythrocytes. Since these cells lack internal structure, the membrane properties and interactions are revealed in the change of cell shape which can be observed under a microscope. There are reports in the literature that nanoparticles can interact with erythrocytes resulting in shape transformations [20,36] and lysis [37]. Some types of nanoparticles cause formation of reactive oxygen species [38], induce platelet activation, aggregation and adhesion [39,40], and increase the risk of thromboembolic disorders [41,42].

In this work we studied the interactions of agglomerated engineered nanoparticles (EPs) with populations of blood cells and with populations of GUVs. We selected two different types of EPs that are commonly present in the environment and have the potential to enter the body and the circulation system, namely TiO_2_ and ZnO EPs.

## Results

**Figure 1:**
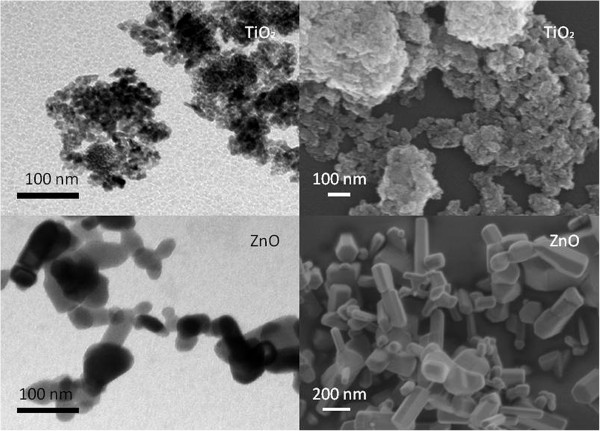
**TiO**_**2**_**and ZnO agglomerated engineered nanoparticles (dissolved in 0.3M glucose solution) imaged by transmission electron microscopy (A,C) and by scanning electron microscopy (B,D).** Concentration of nanoparticles was 10 μg/ml.

**Figure 2:**
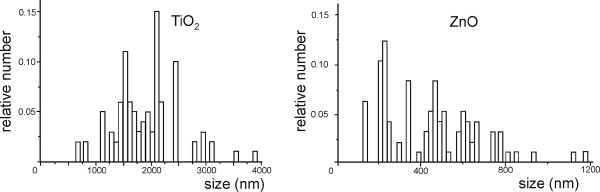
**Relative number of agglomerated engineered nanoparticles distributed over size.** The height of the column represents the number of agglomerated engineered nanoparticles pertaining to a given size interval, divided by the number of all agglomerated engineered nanoparticles measured. Left: the relative number of glucose-suspended TiO_2_ agglomerated engineered nanoparticles, right: the relative number of glucose-suspended ZnO agglomerated engineered nanoparticles, as measured by the dynamic light scattering method.

Figures 1 and 2 show TiO_2_ and ZnO EPs imaged with scanning and transmission electron microscopes and their size distributions, respectively. The populations of TiO_2_ and ZnO EPs in glucose solution as measured with dynamic light scattering (DLS) method were heterogeneous in size (Table 1), with large aggregates present in both suspensions tested. DLS measurements of TiO_2_ and ZnO EP suspensions in PBS citrate could not be performed due to strong agglomeration and consequently fast sedimentation of the EPs.

**Table 1:** Characteristic properties of population of suspensions of agglomerated engineered nanoparticles

**Engineered nanoparticles**	**pH**	**Zeta potential[mV]**	**Stability behaviour**	**Size(measured by DLS)**
TiO_2_ in PBS- citrate	7,9	−26	Incipient instability	
ZnO in PBS- citrate	7,9	−29	Incipient instability	
TiO_2_ in 0,3 M glucose	4,4	−22	Incipient instability	600-4000 nm (average 1820 nm)
ZnO in 0,3 M glucose	6,6	−15	Incipient instability	100 – 1200 nm (average 431 nm)

The DLS measurements of TiO_2_ and ZnO EP suspensions in glucose could be performed, but the DLS measurements of TiO_2_ and ZnO EP suspensions in PBS citrate could not be performed due to fast sedimentation.

When dissolved in PBS or glucose, TiO_2_ EPs and ZnO EPs were found to be mildly negatively charged as they displayed negative zeta potentials (Table 1). Measurements of tested suspensions indicated incipient instability. ZnO EPs in PBS are the least prone to coalesce which is in agreement with the measured size of the EPs (Figure 2, Table 1).

**Figure 3:**
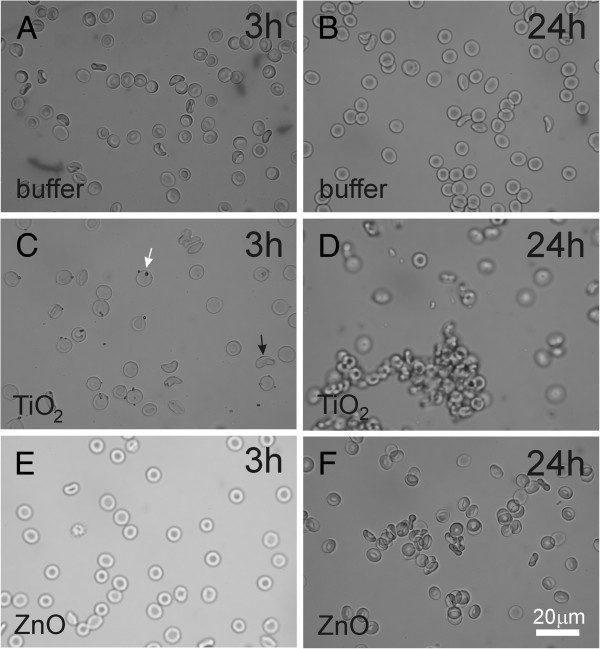
**Effect of PBS-citrate (control) (A, B), TiO_2_ (C, D) and ZnO (E, F) agglomerated engineered nanoparticles on populations of washed human erythrocytes as observed by phase contrast optical microscopy.** Agglomerated engineered nanoparticles were suspended in phosphate buffer saline.

Figure 3 shows the effect of EPs on washed human erythrocytes as observed by phase contrast optical microscope. The time of incubation of the erythrocytes with EPs is indicated in the figure. Incubation with PBS-citrate showed no effect after 3 hours or after 24 hours (Figure 3A, B). TiO_2_ EPs adhered to the erythrocyte membrane after 3 hours (Figure 3C), while after 24 hours clusters of erythrocytes were formed (Figure 3C). ZnO EPs showed no effect after 3 hours (Figure 3E), but after 24 hours clustering of erythrocytes and adhesion of EPs to erythrocytes was observed (Figure 3F).

**Figure 4:**
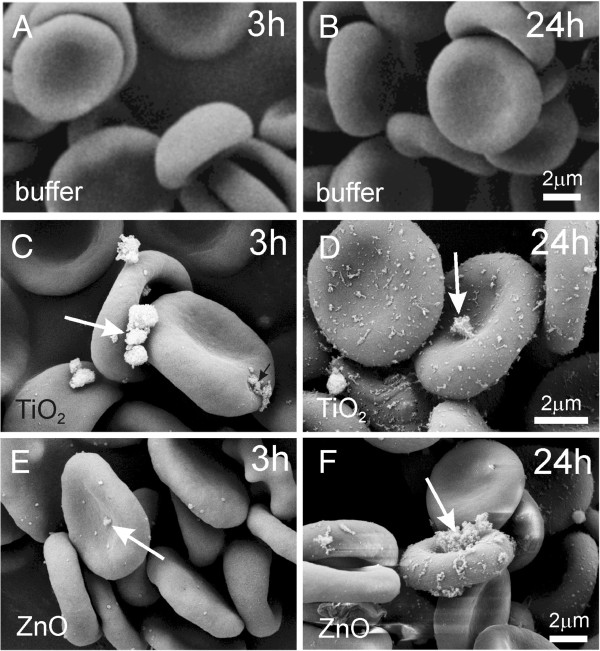
**Effect of PBS-citrate (control) (A, B), TiO_2_ (C, D) and ZnO (E, F) agglomerated engineered nanoparticles on washed human erythrocytes as observed by scanning electron microscopy.** Agglomerated engineered nanoparticles were suspended in phosphate buffer saline. Arrows point to very large agglomerates of nanoparticles.

**Figure 5:**
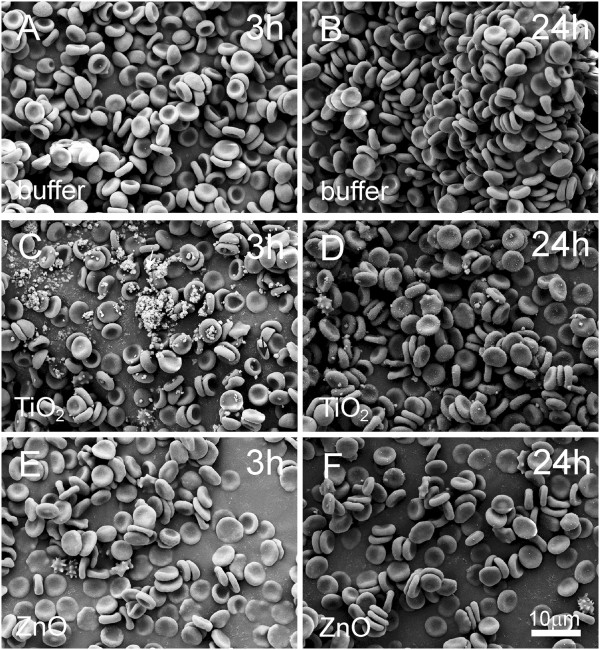
**Effect of PBS-citrate (control) (A, B), TiO_2_ (C, D) and ZnO (E, F) agglomerated engineered nanoparticles on populations of washed human erythrocytes as observed by scanning electron microscopy.** Agglomerated engineered nanoparticles were suspended in phosphate buffer saline.

Figures 4 and 5 show the interaction between EPs and washed human erythrocytes as observed by scanning electron microscopy (SEM). TiO_2_ and ZnO EPs adhered to the membrane (Figure 4C-F). Singular echinocytes could be observed in samples treated with EPs (Figure 5C-F), but not in control samples (A, B).

**Figure 6:**
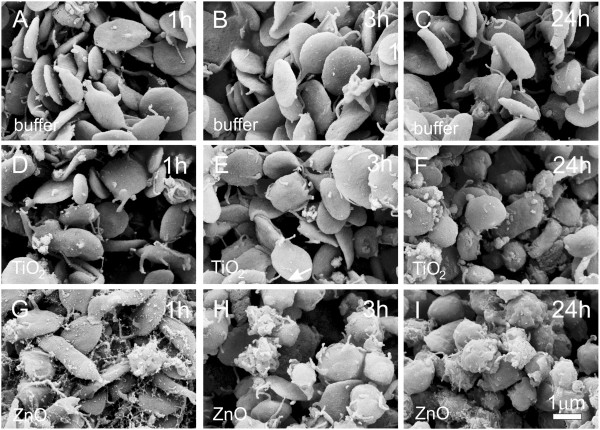
**Effect of PBS-citrate (control) (A-C), TiO_2_ (D-F) and ZnO (G-I) agglomerated engineered nanoparticles on human platelet rich plasma as observed by scanning electron microscopy.** Samples were incubated with agglomerated engineered nanoparticles dissolved in phosphate buffer saline for 1 hour (**A**,**D**, **G**), 3 hours (**B**, **E**, **H**) and 24 hours (**C**, **F**, **I**).

Figure 6 shows the effect of EPs on platelet-rich plasma as observed by SEM. Mostly thin disc-like shapes characteristic of resting platelets can be seen in control samples and in samples incubated with TiO_2_ EPs for 1 hour and for 3 hours. Incubation with TiO_2_ EPs for 24 hours and incubation with ZnO EPs caused transformation to rounded shapes and formation of thin tubular protrusions characteristic of activated platelets. The effect was stronger with longer incubation times. 

**Figure 7:**
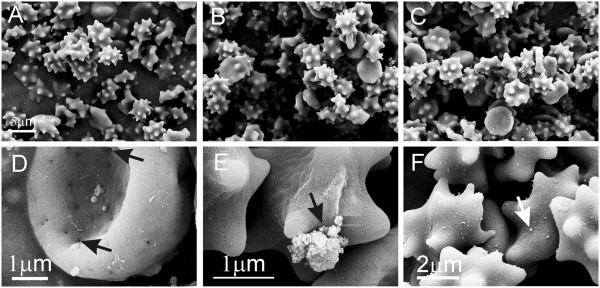
**Effect of PBS-citrate (control), TiO_2_ and ZnO agglomerated engineered nanoparticles on washed canine erythrocytes.** Effect of PBS-citrate (control) (**A**), TiO_2_ (**B**) and ZnO (**C**) agglomerated engineered nanoparticles on populations of washed canine erythrocytes. An erythrocyte with pores (pores are marked by black arrows) in an untreated sample (**D**). Binding of agglomerates to washed canine erythrocytes: large TiO_2_ agglomerate (marked by a black arrow) (**E**) and numerous ZnO aggregates on the surface of echinocyte (marked by a white arrow) (**F**). Agglomerated engineered nanoparticles were suspended in phosphate buffer saline.

Figure 7 shows washed erythrocytes of a healthy dog after incubation with PBS-citrate (A), TiO_2_ EPs (B) and ZnO EPs (C) for 1 hour as observed by SEM. Echinocytes were formed both in control samples and in samples treated with EPs but we observed no difference in populations of erythrocytes between the samples. Pores (marked by an arrow) in the membrane of the untreated erythrocyte (D) and adhesion of a larger TiO_2_ aggregate (E, black arrow) and of numerous ZnO aggregates (F, white arrow) could be observed. As echinocytic shape of erythrocytes was present in test samples and in control sample, it was not ascribed to the effect of EPs.

**Figure 8:**
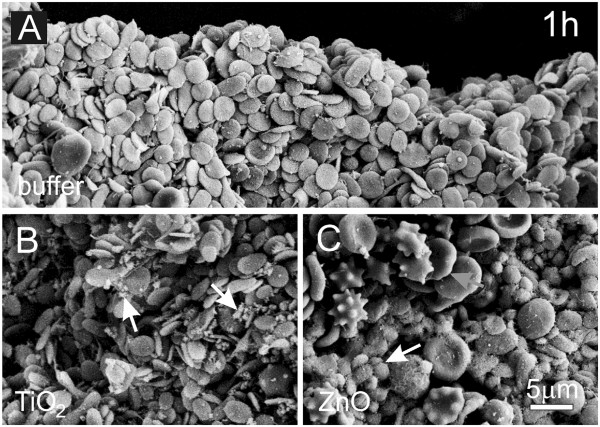
**Effect of PBS-citrate (control) (A), TiO_2_ (B) and ZnO (C) agglomerated engineered nanoparticles on canine platelet-rich plasma.** Platelet-rich plasma was incubated with agglomerated engineered nanoparticles dissolved in phosphate buffer saline for 1 hour. Activated platelets (white arrow) and larger number of erythrocytes (gray arrow) including echinocytes can be seen in the sample incubated with ZnO (**C**).

Figure 8 shows platelet-rich plasma of a healthy dog after incubation with PBS-citrate (A), TiO_2_ EPs (B) and ZnO EPs (C) for 1 hour as observed by SEM. Resting platelets and singular residual erythrocytes were found in samples incubated in buffer or in a suspension of TiO_2_ EPs. Aggregated TiO_2_ EPs are visible in the sample incubated with TiO_2_ (white arrows, B). Incubation in a suspension of ZnO EPs caused transformation of disc shaped platelets into a globular form indicating their activation (white arrow, C) while many residual erythrocytes (gray arrow, C) could be observed.

In experiments with GUVs, a given volume of suspension containing GUVs was placed on glass slide under the microscope. After certain time, GUVs in the focal plane at the bottom of the observation chamber were counted. If GUVs keep their integrity their number at the bottom of the chamber increases with time. Namely, since they contain heavier sugar molecules than the surrounding solution they sediment in the gravitation field. But if nanoparticles are present in the suspension, some vesicles are expected to burst. Therefore the test sample would contain less GUVs than the control sample. Table 2 shows the average number of GUVs per micrograph in test samples and in control samples as observed after 20 and 50 minutes of incubation. As expected, in the test and in the control samples the number of detected GUVs increased with time due to sedimentation of GUVs. Incubation with TiO_2_ EPs caused no significant effect on vesicle number after 20 or 50 minutes, i.e. the increase of the number of GUVs in the test and in the control samples was not statistically significantly different after 20 minutes and after 50 minutes. In the samples exposed to ZnO EPs, a considerable (difference = 17%) and statistically significant (*p* < 10^-3^, *P* = 0,93) decrease of the number of GUVs with respect to the control was observed already after 20 minutes indicating that some GUVs had bursted. The effect became stronger after 50 minutes. The difference in number (28%) was statistically very significant (*p* < 10^-7^, *P* = 1) (Table 2).

**Table 2:** Effect of TiO_2_ and ZnO agglomerated engineered nanoparticles EPs on the average number of giant unilamelar phospholipid vesicles (GUVs) per micrograph

**EPs**	***t*[min]**	**GUVs *N***_**test**_ ± **SD**	**GUVs *N***_**control**_ ± **SD**	**Micrographs *M***_**test**_	**Micrographs *M***_**control**_	**Difference in *N***	***p***	***P*(α** = **0,05)**
TiO_2_	20	8,6 ± 3,6	8,7 ± 3,8	151	151	−1%	0,913	0,03
	50	13,4 ± 5,8	14,8 ± 7,0	155	148	−10%	0,465	0,51
ZnO	20	5,2 ± 2,3	6,3 ± 2,8	156	145	−17%	3.10^-4^*	0,93*
	50	6,5 ± 3,5	9,1 ± 4,3	155	144	−28%	3.10^-8^*	1,00*

The table shows the time of incubation (*t*), the average number of GUVs per micrograph of the test and of the control suspensions (*N*_test_ and *N*_control_, respectively) with their standard deviations (SD), the number of harvested micrographs of the test and of the control suspensions (*M*_test_ and *M*_control_, respectively), the difference between the number of GUVs in the test and in the control suspensions, the statistical significance (*p* value) of the t-test subject to the difference between the test and the control suspensions, and the corresponding statistical power *P* at α = 0,05. Asterisks denote that the difference is statistically significant and of sufficient power.

## Discussion

We studied the effect of TiO_2_ and ZnO EPs on washed human and canine erythrocytes, platelet-rich plasma and on GUVs. We found that both TiO_2_ and ZnO EPs caused coalescence of erythrocytes and activation of platelets, while ZnO EPs also caused bursting of GUVs. We found the effect of both kinds of EPs on the shape of washed erythrocytes in man and in the dog to be minor; in humans, EPs induced a change of shape towards echinocytes in only a small number of erythrocytes (Figure 5), while in the dog, echinocytosis was also present in samples of washed erythrocytes incubated with buffer and was therefore not ascribed to the effect of EPs (Figure 7).

The effects of EPs on washed human and canine erythrocytes and platelet-rich plasma were qualitatively the same but quantitative differences could be observed. In the dog, activation of platelets took place after only 1 hour of incubation of platelet-rich plasma with ZnO EPs, while in man, this effect was observed after 3 hours. Activation of platelets was also observed in samples incubated with TiO_2_ EPs (after 24 hours in man). In the dog, we did not incubate blood for more than one hour, which could be the reason why no effect of TiO_2_ EPs on canine platelets was observed. As some effects of engineered nanoparticles are different in different species it is indicated that experiments on other species cannot give information relevant for humans as regards quantitative limits. In vitro methods are suitable for testing the potentially harmful effects of new materials such as engineered nanoparticles since they address basic mechanisms underlying these effects [43].

Experiments with artificial membranes and selected cell types such as blood cells reveal general mechanisms that take place in all cells, it is however yet unclear how likely the studied situation will occur in a cell. Suspensions of GUVs are relatively well controlled but the biological relevance of the conclusions is limited due to the simplicity of the system. On the other hand, platelet-rich plasma better represents the natural system in blood where complex interactions between engineered nanoparticles and plasma constituents take place.

Alterations in the nanoparticles' environment lead to their assembly into aggregates and agglomerates. This could significantly change their biological impact, so the appropriate characterization of nanoparticles is vital for correct interpretation of the toxicological results. Therefore in studies of the biological reactivity of nanoparticles, their primary characteristics (characteristics of individual particles) and their secondary characteristics (characteristics of aggregates and agglomerates) should be taken into account [44].

Larger EPs were observed to interact with the membranes of washed erythrocytes (Figure 4C-F, Figure 7E). If the same particle interacts with the membranes of two cells it can mediate attractive interaction between the erythrocytes by a bridging interaction. However, besides very large EPs (such as the one shown in Figure 4C-F, Figure 7E), the solution contains EPs with a heterogeneous size distribution. Smaller EPs may induce attractive interaction between membranes even without strong binding to the membrane. A possible mechanism of mediating attractive interaction between membranes derives from the spatial distribution of charge within the mediating particles. Plasma proteins bind to EPs [19] which may affect the interaction of EPs with cells. Also EP-protein complexes may have an internal charge distribution which differs considerably from the distribution within bare EPs. In general, the structure of phospholipid headgroups is multipolar, so the headgroup interface is a source of electric field. Rearrangement of ions in solution in the vicinity of the interface screens the field and creates a gradient [45] in which particles with internal distribution of charge are free to distribute so as to minimize their free energy [46]. This effect is especially energetically favourable when the mediating particles are orientationally ordered between two interacting membranes separated by a small distance (of the order of nanometers) while it is stronger if the charges within the particles are separated by a larger distance [47]. The equilibrium configuration of membranes and mediating molecules appears as adhesion between membranes.

Plasma contains proteins that mediate attractive interaction between membranes [47-50]. EPs may enhance the mediating effect of proteins by forming protein-EP complexes with extended charge distribution and exposure of positively charged domains that favour interaction with the negatively charged glycolipid coat of the erythrocyte membrane.

We did not observe adhesion of EPs on platelets, even though the effect of engineered nanoparticles on platelets induced an activation which is functionally important. It is possible that EPs undergo changes during the incubation period such as decaying into smaller particles and releasing ions into the solution. This influences the osmotic equilibrium, pH equilibrium, screening of the electric field created by charged species and induces chemical changes such as formation of deleterious oxidised species. Divalent ions were found to activate platelet aggregation as the addition of their chelators EDTA and citrate inhibits their effect [51]. Ions of transition elements (Ni, Zn and Mn) cause even faster platelet aggregation than ions of alkaline earth metals (Mg and Ca) [52]. Zn ions can activate protein kinase C and enhance fibrinogen receptor exposure on the surface of platelets stimulated by ADP [53].

On interaction of EPs with the erythrocyte membrane, we observed no perturbation of the membrane curvature due to the adhesion of EPs (Figure 4C-F). However, we observed defects (pores) in the membrane of untreated canine erythrocytes (Figure 7D) and platelets (not shown) which seem large enough to facilitate transmembrane transport of nano-sized (but not micro-sized) EPs. This is in agreement with previous results [21] where it was reported that agglomerates larger than 0,2 μm were found attached to the erythrocyte membrane, whereas smaller aggregates and single particles were found within cells. It was suggested that neither endocytosis which is based on vesicle formation, nor any actin-based mechanisms are likely to account for nanoparticle translocation into the cell [21] but that smaller EPs probably cross the membrane through pores or ion channels [54]. This is in agreement with our observations that no ghosts or haemoglobin-depleted erythrocytes were seen in the erythrocyte samples.

The effects of EPs on GUVs are important for understanding the potential effects of EPs on biological membranes as phospholipid bilayer is a backbone of the biological membrane. Limited biological relevance of the results of experiments with GUVs (due to simplicity of the system) does not mean that these results have no value for understanding effects on biological membranes. It is indicated that ZnO (but not TiO_2_) can cause rupture of the membrane if it can get close to the phospholipid bilayer (Table 2). In biological membrane such situations can be created for example in membrane budding where lateral redistribution of membrane constituents takes place [55]. Molecules in the outer membrane layer that act as spacers and prevent adhesion of cells to each other may be depleted in the neck that forms between the mother cell and the bud. Namely in the neck the curvature of the membrane is negative which is unfavorable for molecules with substantial parts protruding into the external solution.

The basic paradigm of toxicology indicates that the effect of the substance is dose dependent. However, this paradigm is only partly relevant for nanoparticles. Instead, nanoparticles follow a nano-specific mode of action which is not yet completely understood. The behaviour of nanoparticles in liquid media is poorly predicted due to the fact that they aggregate and agglomerate already in exposure to media as well as when they enter a biological system. Some authors report that suspensions of nanoparticles with higher concentrations contain larger aggregates and thus exhibit lower toxic potential ([56] and references therein). In addition, aggregates adhere to surfaces outside or inside organisms and may therefore cause effects without even entering cells. Up to now, no unified directions exist how to test the effects of nanoparticles and how to use the data in human or environmental risk assessment. It is generally accepted that the toxicological tests that are used to assess the toxicity of non-nanosized substances should be modified.

## Conclusions

We found that addition of TiO_2_ and ZnO EPs to erythrocytes suspended in PBS-citrate caused adhesion of EPs to the membranes of erythrocytes and erythrocyte coalescence. Addition of TiO_2_ and ZnO EPs induced activation of platelets. This indicates that EPs could be potentially prothrombogenic. ZnO (but not TiO_2_) EPs suspended in glucose solution caused a significant decrease of GUVs indicating that ZnO EPs (but not TiO_2_ EPs) could cause membrane rupture. Blood cells and phospholipid vesicles are convenient systems for the study of membrane properties and their interactions with various substances. As knowledge on the relevant mechanisms is as yet rudimentary, we suggest that further studies using these systems should be performed.

## Methods

### Chemicals

The phospholipid 1-Palmitoyl-2-Oleoyl-sn-Glycero-3-Phosphocholine (POPC) and cholesterol were purchased from Avanti Polar Lipids, Inc. (Alabaster, Al, USA). Sucrose and glucose were purchased from Sigma–Aldrich (Steinheim, Germany).

### Engineered particles

Titanium (IV) oxide nanopowder (TiO_2_), supplied as a powder of anatase crystalline structure, with guaranteed 99,7% purity, and zinc oxide nanopowder (ZnO) were purchased from Sigma Aldrich (Steinheim, Germany).

### Preparation of engineered particle suspensions

For experiments with washed erythrocytes or platelet-rich plasma, stock suspensions of EPs were prepared in phosphate buffer saline with trisodium citrate (PBS –citrate: 137 mM NaCl, 2,7mM KCl, 10 mM Na_2_HPO_4_x2H_2_O, 2 mM KH_2_PO_4_, 10,9 mM Na_3_C_6_H_5_O_7_) with a concentration of 5 mg/ml. For experiments with GUVs, stock suspensions of TiO_2_ and ZnO EPs were prepared in 0,3 M glucose with a concentration of 1 mg/ml.

### Characterization and imaging of engineered particle suspensions

Characterization of EPs (primary characteristics of EPs) and their suspensions (secondary characteristics of EPs) were assessed by scanning and transmission electron microscopy (SEM and TEM, respectively), dynamic light scattering (DLS) and zeta potential (ζ) measurements. For TEM, water suspension of EPs was applied on a copper-grid-supported, perforated, transparent carbon foil at room temperature. TEM imaging of EPs was performed using a JEOL 2100 transmission electron microscope (Tokyo, Japan) operated at 200 kV. SEM imaging of EPs in PBS-citrate was performed using a LEO Gemini 1530 (LEO, Oberkochen, Germany) scanning electron microscope.

Dispersed TiO_2_ and ZnO EPs dissolved in 0,3 M glucose (10 μg/ml) were inspected by DLS using a 3D DLS-SLS spectrometer (LS Instruments, Fribourg, Switzerland). Due to very fast sedimentation of both TiO_2_ and ZnO EPs, DLS measurements in PBS could not be performed. The zeta potential (ζ) of TiO_2_ and ZnO EPs suspensions in both media, PBS-citrate and in 0,3 M glucose, were measured at the original concentrations and the pH values of the suspensions using a ZetaPals instrument (Brookhaven Instruments Corporation, Holtsville, NY, USA).

### Blood sampling

Blood was collected from the authors and from 3 healthy pet dogs owned by members of the staff of Prva K Klinika za male živali d.o.o. Ljubljana. 2,7 ml tubes containing 270 μl trisodium citrate at a concentration 0,109 mol/l were used. All the dogs weighed over 15 kg so the volume of the blood sample (2,7 ml) was small compared to the whole volume of the animal. Blood was collected by vein puncture into evacuated tubes (BD Vacutainers, Becton Dickinson, CA) using a 21-gauge needle (length 70 mm, inner radius 0,4 mm) (Microlance, Becton Dickinson, NJ, USA). Sampling was performed according to the Declaration of Helsinki. Informed consent was obtained from all human participants in this study and written consent to take blood from the dogs was obtained from their owners. The study as regarding both human and animal samples was approved by the Slovenian National Medical Ethics Committee, No 117/02/10. No adverse effects on human and animal donors’ health due to sampling were observed.

### Preparation of blood cells for microscopy

Blood was processed within 1 hour of sampling. Blood was centrifuged in a Centric 400R centrifuge (Domel d.o.o., Železniki, Slovenia) at 150 g and 37°C for 10 minutes (human) and at 50 g and 37°C for 15 minutes (dog) to separate erythrocytes from platelet-rich plasma. Erythrocytes were repeatedly washed with PBS citrate by centrifugation at 1550 g and 37°C for 10 minutes. Washed erythrocytes or platelet-rich plasma were aliquotted into equal parts (50 μl in case of erythrocytes and 200 μl in case of platelet-rich plasma). TiO_2_ or ZnO EPs suspended in PBS-citrate (or PBS-citrate alone for the control) was added to aliquot in a v/v ratio of 2:1 in the case of erythrocytes and 3:1 in the case of platelet-rich plasma, 3 and 2 units corresponding to erythrocytes and platelet-rich plasma, respectively. After incubation the samples were fixed in 0,1% glutaraldehyde, incubated for another hour at room temperature and centrifuged at 1550 g and 37°C for 10 minutes. The supernatant was exchanged with PBS-citrate, samples were vortexed, centrifuged at 1550 g and 37°C for 10 minutes and fixed in 2% glutaraldehyde for an hour.

### Phase contrast microscopy of erythrocytes

5 μl of erythrocyte suspension was placed in an observation chamber composed of two cover glasses which were glued together with nail polish. Samples were observed under an Olympus GWB BH-2 (Olympus Corporation, Tokyo, Japan) microscope with phase contrast optics at an objective magnification of 400×. Images were taken with a Canon EOS 450D digital camera (Canon Inc., Tokyo, Japan).

### Scanning electron microscopy of blood cells

Fixed samples were washed by exchanging supernatant with citrated PBS and incubated for 20 minutes at room temperature. This procedure was repeated 4 times while the last incubation was performed overnight at 8°C. Samples were then post-fixed for 60 min at 22°C in 1% OsO_4_ dissolved in 0,9% NaCl, dehydrated in a graded series of acetone/water (50%-100%, v/v), critical-point dried, gold-sputtered, and examined using a LEO Gemini 1530 (LEO, Oberkochen, Germany) scanning electron microscope.

### Preparation of giant unilamelar phospholipid vesicles

The electroformation of GUVs was performed at room temperature. 40 μl of the lipid mixture of POPC (80%, v/v) and cholesterol (20%, v/v), both dissolved in 2:1 chloroform/methanol mixture, was spread over two platinum electrodes and the solvent was allowed to evaporate in low vacuum for two hours. The electrodes were then placed in the electroformation chamber (a 2 ml Eppendorf cup), filled with 2 ml of 0,3 M sucrose solution. An alternating electric field was applied as described in [55]. After electroformation, 600 μl of 0,3M sucrose solution containing GUVs was added to 1 ml of 0,3M glucose solution.

### Experiments with giant unilamelar phospholipid vesicles and engineered particles

Experiments with the two types of EPs, TiO_2_ and ZnO, were performed separately, but following the same procedure. After electroformation, the suspension of GUVs in sucrose/glucose solution was mixed by turning the Eppendorf cup upside down (five times). The suspension was diluted (300 μl of 0,3M glucose solution was added to 100 μl of original vesicle solution) in order to obtain the desired concentration of GUVs, which was found convenient according to preliminary experiments. Subsequently, the diluted suspension was aliquotted into 4 vials, (81 μl in each vial). Duplicates of test suspension of EPs (TiO_2_ or ZnO, both dissolved in 0,3M glucose solution with a concentration of 100 μg/ml) and duplicates of control suspension (0,3M glucose solution) were added to GUV suspensions in a volume ratio of 9:1 (GUVs: test suspension) to reach the final concentration of EPs of 10 μg/ml. Vials were turned upside down five times to mix the test suspension with the GUV suspension. The samples (70 μl each) were separately transferred into four CoverWellTM Perfusion chambers PC4L-0,5 (Grace Bio-Labs Sigma-Aldrich, Steinheim, Germany) to create duplicates for the test sample and duplicates for the control sample.

The observation chambers with samples were mounted to the inverted phase contrast light microscope (Nikon Eclipse TE2000-S, Tokyo, Japan). After pre-defined durations of incubation (20 and 50 minutes), GUV populations in each chamber were recorded by a Sony CCD video camera module, model: XC -77 CE (Minato, Japan). These recordings consisted of two video sequences each approximately 2 minutes long. During recording, the object glass was moved to capture images of thousands of GUVs. Using image processing algorithms, the video sequences were transformed into large mosaics [57] where each mosaic contained all the vesicles acquired within the sample. GUVs in mosaics were segmented using a computer-aided approach [58].

### Statistical analysis

Here we introduced improvements to the analysis of GUV populations. The mosaics were separated into images (micrographs) with the size of a single field of view with the microscope at a given magnification. Using the GUVs’ locations inside the mosaics, all vesicles were associated with the appropriate micrograph, allowing us to extract information on vesicle density throughout the micrographs composing the mosaic.

The numbers of GUVs in a micrograph were averaged over all micrographs within the test and control samples. Methods of descriptive statistics were used to compare the test samples and the respective control samples. We used a two-sided pooled t-test with equal variance. A probability of the t-test below 0,05 was considered statistically significant. Power analysis was performed in order to validate the size of the samples. A power larger than 0,9 at α = 0.05 indicated a sample of proper size. The software used for statistical analysis was from R Development Core Team: R: A language and environment for statistical computing. R Foundation for Statistical Computing, Vienna, Austria. 2008 ISBN 3-900051-07-0, URL http://www.R-project.org.

## Abbreviations

EPs: Agglomerated engineered nanoparticles; GUVs: Giant unilamelar phospholipid vesicles; POPC: Palmitoyloleoyl phosphatidylcholine; TiO_2_: Titanium dioxide; ZnO: Zinc oxide; SEM: Scanning electron microscope; TEM: Transmission electron microscope; DLS: Dynamic light scattering.

## Competing interests

Authors declare no competing interests.

## Authors’ contributions

MŠ participated by blood sampling, experiments with blood cells, design of the study, writing the manuscript and its critical reading, BD prepared EPs suspensions, performed experiments with GUVs, prepared recorded GUVs for statistical analysis and critically read the manuscript, VŠ and RŠ performed experiments with blood cells, prepared EPs and blood samples for SEM and critically read the manuscript, VŠ also performed imaging of EPs and blood cells by phase contrast microscopy and SEM. JZ participated in experiments with GUVs, in improving the statistical analysis of GUVs, performed the statistical analysis and critically read the manuscript. DE participated in improving the method for statistical analysis of GUVs and critically read the manuscript. DM characterized EPs by TEM imaging, DLS and zeta potential measurements and critically read the manuscript. HH participated in the design of the study, performed experiments with blood cells and their imaging with phase contrast microscopy and SEM and critically read the manuscript. DD participated in the design of the study, writing the manuscript and its critical reading. VKI participated in design of the study, writing the manuscript and its critical reading and in improving the method for statistical analysis of GUVs. All authors read and approved the final manuscript.
